# Efficacy and safety of electroacupuncture combined with Suanzaoren decoction for insomnia following stroke: study protocol for a randomized controlled trial

**DOI:** 10.1186/s13063-021-05399-y

**Published:** 2021-07-24

**Authors:** Hui-lian Huang, Song-bai Yang, Zhi-gang Mei, Ya-guang Huang, Mao-hua Chen, Qun-li Mei, Hua-ping Lei, Qing-xian Mei, Jian-hua Chen

**Affiliations:** 1 School of Basic Medical Sciences, Zhejiang Chinese Medical University, Zhejiang, 310053 Hangzhou China; 2 College of Traditional Chinese Medicine, China Three Gorges University & Yichang Hospital of Traditional Chinese Medicine, Yichang, 443003 Hubei China; 3 Key Laboratory of Hunan Province for Integrated Traditional Chinese and Western Medicine on Prevention and Treatment of Cardio-Cerebral Diseases, Hunan University of Chinese Medicine, Changsha, 410208 Hunan China; 4 Medical College of China Three Gorges University, Yichang, 443002 Hubei China; 5 Affiliated Renhe Hospital of China Three Gorges University, Yichang, 443001 Hubei China

**Keywords:** Electroacupuncture, Suanzaoren decoction, Insomnia, Stroke, Randomized controlled trial

## Abstract

**Background:**

Insomnia is a common but frequently overlooked sleep disorder after stroke, and there are limited effective therapies for insomnia following stroke. Traditional Chinese medicine (TCM), including acupuncture and the Chinese herbal medication (CHM) Suanzaoren decoction (SZRD), has been reported as an alternative option for insomnia relief after stroke in China for thousands of years. Here, this study aims to investigate the efficacy and safety of electroacupuncture (EA) in combination with SZRD in the treatment of insomnia following stroke.

**Methods:**

A total of 240 patients with post-stroke insomnia will be included and randomized into four groups: the EA group, SZRD group, EA & SZRD group, and sham group. The same acupoints (GV20, GV24, HT7, and SP6) will be used in the EA group, EA & SZRD group, and sham group, and these patients will receive the EA treatment or sham manipulation every other day for 4 consecutive weeks. SZRD treatments will be given to participants in the SZRD group and EA & SZRD group twice a day for 4 consecutive weeks. The primary outcome measures include Pittsburgh Sleep Quality Index scores and polysomnography. Secondary outcome measures include the Insomnia Severity Index, the National Institutes of Health Stroke Scale, the Hospital Anxiety and Depression Scale, brain magnetic resonance imaging, functional magnetic resonance imaging, and nocturnal melatonin concentrations. The primary and secondary outcomes will be assessed at baseline (before treatment), during the 2nd and 4th weeks of the intervention, and at the 8th and 12th weeks of follow-up. Safety assessments will be evaluated at baseline and during the 4th week of the intervention.

**Discussion:**

This study will contribute to assessing whether the combination of these two therapies is more beneficial for post-stroke insomnia than their independent use, and the results of this clinical trial will improve our understanding of the possible mechanisms underlying the effects of combination therapies.

**Trial registration:**

Chinese Clinical Trials Register ChiCTR2000031413. Registered on March 30, 2020

## Administrative information

**Table:** 

Title {1}	Efficacy and safety of electroacupuncture combined with Suanzaoren decoction for insomnia following stroke: protocol for a randomized controlled trial
Trial registration {2a and 2b}.	Chinese Clinical Trials Register, ID: ChiCTR2000031413. Registered on March 30, 2020.
Protocol version {3}	Date January 16, 2020 and version 2.0
Funding {4}	The study was supported by Yichang Municipal Commission of Science and Technology (A19-301-42)
Author details {5a}	1. School of Basic Medical Sciences, Zhejiang Chinese Medical University, Hangzhou, Zhejiang 310053, China 2. College of Traditional Chinese Medicine, China Three Gorges University & Yichang Hospital of Traditional Chinese Medicine, Yichang, Hubei 443003, China 3. Key Laboratory of Hunan Province for Integrated Traditional Chinese and Western Medicine on Prevention and Treatment of Cardio-Cerebral Diseases, Hunan University of Chinese Medicine, Changsha, Hunan 410208, China 4. Medical College of China Three Gorges University, Yichang, Hubei 443002, China 5. Affiliated Renhe Hospital of China Three Gorges University, Yichang, Hubei 443001, China
Name and contact information for the trial sponsor {5b}	College of Traditional Chinese Medicine, China Three Gorges University & Yichang Hospital of Traditional Chinese Medicine, Yichang, Hubei 443003, China
Role of sponsor {5c}	The study funder has no role in the study design, data collection and management, and manuscript writing publication

## Introduction

### Background and rationale {6a}

Stroke is the second leading cause of death and a major cause of disability worldwide. It has become one of the major contributors to the increased prevalence, incidence, and mortality of vascular disease [1]. Over 2 million people suffer a stroke in China every year [2]. Insomnia is the most common disorder of the sleep-wake cycle, affecting approximately 50% of stroke survivors [3]. Insomnia is defined as repeated difficulties in initiating or maintaining sleep or early awakening accompanied by daytime functional impairment [4]. In some cases, insomnia that develops in young adulthood may be a higher risk factor for stroke than insomnia that develops later [5]. On the other hand, insomnia may directly result from cerebral ischaemia or anoxia during stroke or environmental conditions in inpatient wards or stroke-related medical conditions such as pain, depression, infections, and drug-induced side effects [6, 7]. Insomnia following a stroke can deteriorate cognitive and physical function, contributing to a worse outcome during rehabilitation [8]. Currently, the American Academy of Sleep Medicine guideline recommends cognitive behaviour therapy as a treatment for insomnia, but it is not easily or widely available [9]. Meanwhile, the side effects of hypnotic medications, such as drowsiness, fatigue, and dizziness, limit their usage in patients with post-stroke insomnia [10]. Thus, owing to the lower side effects and holistic efficacy, complementary and alternative therapies, including acupuncture and/or Chinese herbal medication (CHM), have been incorporated into the treatment of insomnia following stroke worldwide [11].

Electroacupuncture (EA), a new form of acupuncture where a small electric current is passed between pairs of acupuncture needles, has shown positive effects on several biological parameters, such as enhancing cognitive function, learning, memory, and sleep following stroke [12]. EA beneficially regulates a healthy mental state by stimulating some special meridian points [13]. Based on theories in traditional Chinese medicine (TCM), the acupoints of the *Governor meridian* (Dumai in Chinese) and the *Shaoyin meridian* (Shaoyin Jing in Chinese) are the most common acupoints used for the treatment of stroke and its complications [14]. The distal acupoints are commonly matched with the local acupoints based on the mechanism of the disease in accordance with the theory of TCM. A systematic review of the effects of acupuncture on insomnia after stroke has shown potential benefits in the treatment of insomnia and for improving patients’ sleep quality [15].

Chinese medicinal herbs and formulas have also been widely used to treat some neurological diseases, such as stroke, depression, and insomnia, in China and some Asian countries for thousands of years [16]. In recent decades, some of them have also been recommended worldwide to alleviate insomnia [17]. Suanzaoren decoction (SZRD), a well-known Chinese medicinal formula for insomnia, is a combination of five CHMs based on TCM theory and includes Suanzaoren (Ziziphi Spinosae Semen), Fuling (Poria cocos), Chuanxiong (Ligusticum wallichii), Zhimu (Anemarrhenae), and Gancao (Glycyrrhiza) [18]. A previous meta-analysis showed that SZRD solely or combined with other treatments could promote sleep quality and prolong sleep duration [19].

Therefore, combining EA with SZRD into a treatment method can potentially be used in the clinical therapy of post-stroke insomnia patients.

## Objectives {7}

Although some studies have shown acupuncture combined with SZRD is more beneficial for insomnia [20, 21], whether EA combined with SZRD is more effective and safer for insomnia after stroke needs more clinical and experimental evidence. Therefore, we will combine EA with SZRD as a clinical treatment method for patients with post-stroke insomnia. We aim to assess whether the combination therapies are more effective and safer for insomnia after stroke than independent usage of the two treatments and to preliminarily uncover the possible mechanisms underlying the effect of the combination therapy.

## Trial design {8}

The study is designed as a 12-week, single-centre, randomized, single-blind, controlled trial. Study participants will be included according to the inclusion and exclusion criteria. Before starting the treatment, all eligible patients will undergo a standard neuroimaging examination (brain magnetic resonance imaging and functional magnetic resonance imaging), a baseline evaluation of sleep quality, neurological deficits, anxiety and depression, and measurement of nocturnal melatonin concentrations. Then, subjects will be equally randomized into four groups: the EA group, SZRD group, EA & SZRD group, and sham group. Patients in the EA group and sham group will receive EA stimulation or sham EA every other day for 4 consecutive weeks for a total of 14 sessions. Patients in the SZRD group will only take SZRD granules orally twice a day for 4 consecutive weeks. Patients in the EA & SZRD group will be administrated SZRD granules orally twice a day for 4 consecutive weeks, combined with EA stimulation every other day for 4 consecutive weeks, for a total of 14 sessions. Assessments will be carried out at baseline (before treatment), during the 2nd and 4th weeks of the intervention, and at the 8th and 12th weeks of follow-up.

The trial will be performed according to the principles of the Declaration of Helsinki (Fortaleza version, 2013), the Consolidated Standards of Reporting Trials [22], the Standards for Reporting Interventions in Clinical Trials of Acupuncture [23], and Recommendations for Intervention Trials (SPIRIT) Checklist.

## Methods: participants, interventions, and outcomes

### Study setting {9}

**Fig. 1:**
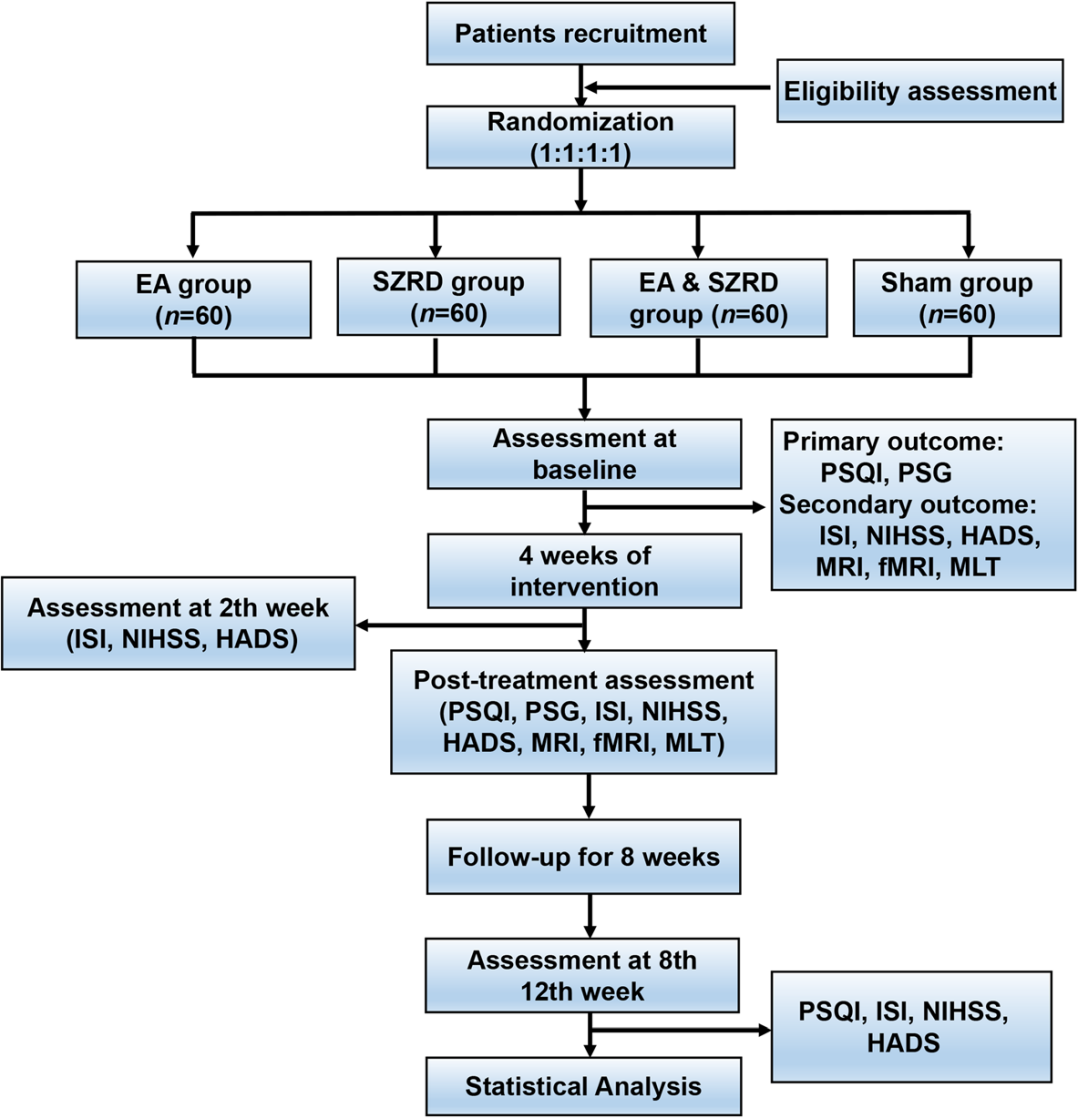
Flow chart. EA, electroacupuncture; SZRD, Suanzaoren decoction; PSQI, Pittsburgh Sleep Quality Index; PSG, polysomnography; ISI, Insomnia Severity Index; NIHSS, National Institutes of Health Stroke Scale; HADS, Hospital Anxiety and Depression Scale; MRI, brain magnetic resonance imaging; fMRI, functional magnetic resonance imaging; MLT, nocturnal concentration of melatonin

A total of 240 patients with post-stroke insomnia will be recruited through advertisements on the WeChat official platform and hospital bulletin boards at the College of Traditional Chinese Medicine, China Three Gorges University & Yichang Hospital of Traditional Chinese Medicine. Written informed consent will be provided by all patients at the time of recruitment. Patients will have an equal chance of being randomly allocated to the EA group, SZRD group, EA & SZRD group, and sham group. Due to the limitations of the intervention methods, the therapists will not be blinded. The assessors will perform the evaluations and analysis of the outcomes at five points (before treatment, during the 2nd and 4th weeks of the intervention, and the 8th and 12th weeks of follow-up). Data management and statistics will be conducted at the Medical College of China Three Gorges University. The flow chart is shown in the SPIRIT Figure (Fig. 1).

### Eligibility criteria {10}

#### Inclusion criteria

Participants who meet the following criteria will be included in the trial:

1. Aged 18–70 years, either sex;
2. Fulfilling the diagnostic criteria of stroke based on the Chinese guidelines for diagnosis and treatment of acute ischemic stroke 2018 [24] or Chinese guidelines for diagnosis and treatment of acute intracerebral haemorrhage 2019 [25];
3. After acute stroke; and
4. Fulfilling the diagnostic criteria of insomnia according to the *Diagnostic and Statistical Manual of Mental Disorders, fifth edition* [4].

#### Exclusion criteria

Participants who meet the following criteria will be excluded from the trial:

1. Had insomnia before stroke;
2. Insomnia by poor sleep conditions [26];
3. Have diagnoses of other sleep disorders, such as narcolepsy, obstructive sleep apnoea syndrome, or restless legs syndrome;
4. Have neurological or psychiatric diseases;
5. Have severe diabetes; hypertension; severe liver, kidney, or thyroid dysfunction; and severe cardiac insufficiency;
6. Are unable to read, understand, and complete the forms or use the rating scales;
7. Have implants that could interfere with EA or a history of hypersensitivity to EA;
8. Had received TCM in the previous 1 month;
9. Had participated in other trials;
10. Are taking sleep-affecting diet or medication; or
11. Are pregnant or preparing for pregnancy.

### Who will take informed consent? {26a}

The principal investigator at Yichang Hospital of Traditional Chinese Medicine will conduct the informed consent process, who will screen the potential participants in accordance with the inclusion and exclusion criteria. Written informed consent will be provided by eligible patients at the time of recruitment.

### Additional consent provisions for collection and use of participant data and biological specimens {26b}

During the intervention period, patients will have the right to withdraw for whatever reason and at whatever time. Therefore, on the consent form, participants are asked if they agree to the use of their data when they choose to withdraw from the trial. Participants will also be asked for permission for the research team to share relevant data with people from the Medical College of China Three Gorges University or Yichang Hospital of Traditional Chinese Medicine. This trial does not involve collecting biological specimens that will be stored.

## Interventions

### Explanation for the choice of comparators {6b}

In this study, we will assess whether EA combined with SZRD is more beneficial for post-stroke insomnia than independent usage of the two treatments. Therefore, EA, SZRD, and sham EA were chosen as comparators in this trial.

### Intervention description {11a}

All manipulators with acupuncturist qualification certificates have independently performed clinical treatments for more than 2 years. The acupuncturists will engage in the clinical training before the intervention to ensure experience with the standard real and sham acupuncture operations and will not be replaced during the trial.

**Fig. 2:**
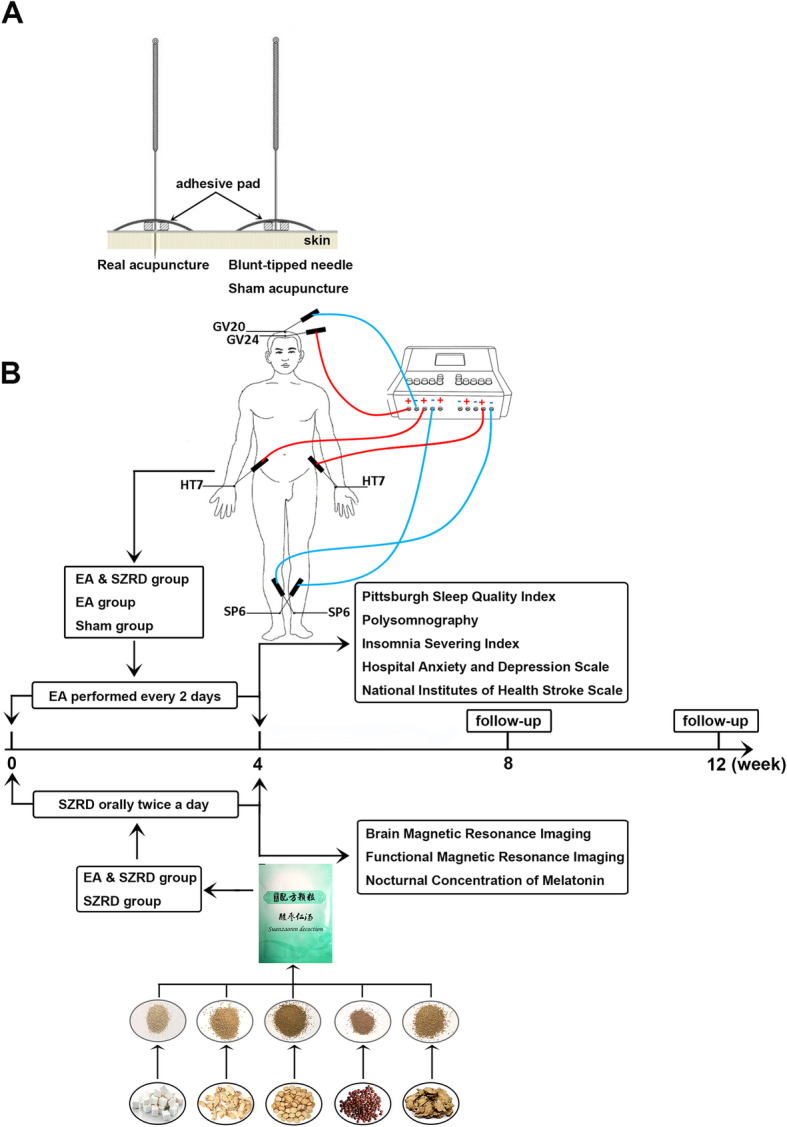
The manipulation of the real and sham acupuncture device and intervention process chart. **A** The manipulation of the real and sham acupuncture. **B** The intervention process chart

Disposable acupuncture needles (0.35 × 25 mm or 0.35 × 40 mm, JiaJian Medical Instrument Co., Ltd, Wuxi, China), sham acupuncture needles with blunt tips (0.30 × 25 mm), G6805-2A EA devices (Huayi Medical Instrument Co., Ltd, Shanghai, China), and SZRD granules (Tcmages Pharmaceutical Co., Ltd, Beijing, China) will be used in this trial. The acupuncturists will use 0.35 × 25 mm needles for acupoints GV20, GV24, and HT7 and 0.35 × 40 mm needles for acupoint SP6. The depth of needle insertion will be 10–25 mm for each acupoint. Participants will receive EA or sham EA treatment every other day for 4 weeks for a total of 14 sessions, and participants receiving SZRD treatment will take 3.4 g SZRD granules orally twice a day for 4 weeks. After the baseline assessment, it will take 12 weeks for a patient to complete the trial, including 4 weeks of treatment and 8 weeks of follow-up (the intervention process chart is shown in Fig. 2B). Before the manual operation, patients will be asked to wear eye masks and lie supine. Each session will last for 30 min. The temperature in the isolated treatment room will be 25 °C (the interventions for all groups are shown in Table 1).

**Table 1:** Intervention parameters of each group

Groups	Interventions			
	Acupoints	EA parameters	Chinese herbs	Treatment sessions
EA & SZRD group	Bilateral HT7, bilateral SP6, GV20, GV24	Continuous wave stimulation; frequency = 10 Hz; current ranges from 1 to 5 mA	Suanzaoren, Zhimu, Fuling, Chuanxiong, Gancao	4 weeks, EA performed every other day, total 14 sessions; SZRD granules orally taken twice a day
EA group	Bilateral HT7, bilateral SP6, GV20, GV24	Continuous wave stimulation; frequency = 10 Hz; current ranges from 1 to 5 mA	No herbs	4 weeks, EA performed every other day, total 14 sessions
SZRD group	No acupoints	No parameters	Suanzaoren, Zhimu, Fuling, Chuanxiong, Gancao	4 weeks, SZRD granules orally taken twice a day
Sham group	Bilateral HT7, bilateral SP6, GV20, GV24 (no need inserting)	Frequency = 0 Hz; current = 0 mA	No herbs	4 weeks, sham EA performed every other day, total 14 sessions

#### EA group

Subjects in this group will receive EA treatment. The following acupoints were selected: local acupoints: *Baihui* (GV20) and *Shenting* (GV24); distal acupoints: bilateral *Shenmen* (HT7) and bilateral *Sanyinjiao* (SP6). After skin disinfection, sterile adhesive pads will first be fixed on the acupoints, and the acupuncture needles will be inserted through the adhesive pads into the skin. Needle insertion will follow an angle of 30° along the anterior-posterior midline for GV20 and GV24. Perpendicular punctures will be used for HT7 and SP6. Acupuncturists will manipulate the needle until patients experience the *de qi* sensation (manifesting as soreness, fullness, heaviness, and numbness when lifting, thrusting, or rotating the needle). There are three sets of electrodes connected. One set of two electrodes of the EA device are connected at GV20 and GV24. Another two sets of electrodes are connected at the ipsilateral HT7 and SP6. The electroacupuncture intervention will apply 10-Hz continuous waves, and the current will range from 1 to 5 mA. The intensity will be adjusted based on the tolerance of each patient. Each session will last 30 min and be performed every other day for 4 successive weeks, for a total of 14 sessions.

#### SZRD group

Subjects in this group will receive SZRD treatment. SZRD consists of 5 herbs, including Suanzaoren, Fuling, Zhimu, Chuanxiong, and Gancao, in the following concentrations: 41%, 20%, 20%, 13%, and 6%, respectively. Patients will be administered SZRD granules in one bag (3.4 g per bag) dissolved in boiling water two times daily for 4 successive weeks.

#### EA & SZRD group

Subjects in this group will receive the EA and SZRD treatments. The procedures for the EA and SZRD treatments will be the same as those for the EA group and SZRD group. EA will last 30 min in each session, which will be performed every other day for 4 successive weeks, for a total of 14 sessions. SZRD treatment will be given two times daily for 4 successive weeks.

#### Sham group

Subjects in this group will receive sham EA treatment with sham acupuncture needles. The acupoints selected, manipulation procedures, electrode placements, and other treatment settings will be the same as in the EA group, while the effects of *de qi* will not be produced through skin penetration or needle manipulation. Furthermore, all parameters of the electrical stimulation will be set to 0. After retaining the needles for 30 min, acupuncturists will remove all needles using dry cotton balls and press the acupoints to provide patients with the sensation of a ‘real’ needle being withdrawn from the skin. Each session will last 30 min and will be performed every other day for 4 successive weeks.

### Criteria for discontinuing or modifying allocated interventions {11b}

If participants feel that their diseases are not improving, they will be free to withdraw from the clinical study and choose other therapies. Participants who meet the following criteria could be discontinued from receiving the interventions:

1. They develop a serious disease such as heart disease, pneumonia, or stroke during the trial;
2. They receive other treatments for insomnia such as other medicine, moxibustion, or massage, during the study;
3. They experience serious side effects;
4. They refuse to continue to participate in the RCT;
5. They are lost to follow-up; or
6. They do not comply with the study protocol.

### Strategies to improve adherence to interventions {11c}

Frequent follow-up telephone calls and messages on the WeChat platform will be important aspects for improving adherence to interventions.

### Relevant concomitant care permitted or prohibited during the trial {11d}

Given the history of stroke, all participants will continue to take basic Western medications for the secondary prevention of stroke during the study. The name, dose, and usage of the medications will be recorded.

### Provisions for post-trial care {30}

If the condition of a subject worsens after the trial or is accompanied by severe complications or serious adverse reactions, the specialist will take medical measures to deal with the harm based on the subject’s condition. The treatments will be free.

### Outcomes {12}

#### Primary outcomes

The following primary outcomes will help us to assess whether the combination of EA with SZRD is more effective in insomnia in post-stroke patients.

##### Pittsburgh Sleep Quality Index

The PSQI is a self-rating questionnaire scale with 19 self-rated items and five other-rated items measuring sleep quality and disturbances over a period of 1 month [27]. The self-rated items include the following seven subscales: sleep quality, sleep latency, sleep duration, habitual sleep efficacy, sleep disturbances, use of sleeping medication, and daytime dysfunction. Each subscale will be answered by patients on a 0 to 3 response category scale, and the total score of the PSQI can range from 0 to 21 points. A lower score indicates a better quality of sleep. The assessment will be performed at baseline (before treatment) and during the 4th week of the intervention, as well as at the 8th and 12th weeks of follow-up.

##### Polysomnography

PSG (Curative Medical Inc, Santa Clara, CA, USA) is used to objectively estimate sleep [28]. It records quantifiable data during sleep, such as total sleep time, sleep onset latency, waking after sleep onset, rapid eye movement, and non-rapid eye movement sleep. In this study, all patients will be assessed for two nights (one night at baseline and one night at 4 weeks post-treatment) in a specialized sleep laboratory. During the assessment process, the sleep time of participants will be based on their habit. Standard equipment will record sleep conditions from 22:00 to 7:00 for each assessment. All PSG data will be further analysed by Acumen 7 EV 2.11 software.

#### Secondary outcomes

The following secondary outcomes will help us to determine whether participants with post-stroke insomnia in the EA & SZRD group show greater improvements in sleep quality and other related symptoms than the other three groups after the therapies.

##### Insomnia Severity Index

The ISI is a self-report questionnaire designed to assess the degree of insomnia and the associated functional impairments [29]. The total score on the ISI can range from 0 to 28 points. A score of 0 to 7 indicates no clinically significant insomnia. A score of 8 to 14 is regarded as sub-threshold insomnia. A score of 15 to 21 is regarded as clinical insomnia of moderate severity. More than 22 points is regarded as severe clinical insomnia. The assessment will be performed at baseline (before treatment) and during the 2nd and 4th weeks of the intervention, as well as at the 8th and 12th weeks of follow-up.

##### Hospital Anxiety and Depression Scale

The HADS is a brief assessment used to measure current anxiety and depressive symptoms in a non-psychiatric hospital patient [30]. The HADS comprises two independent seven-item subscales for anxiety and depression. More than 11 points on either subscale indicate the probable presence of the mood disorder. The assessment will be performed at baseline (before treatment) and during the 2nd and 4th weeks of the intervention, as well as at the 8th and 12th weeks of follow-up.

##### National Institutes of Health Stroke Scale

The NIHSS is a stroke-specific quantitative scale used to assess neurological deficits [31]. Each participant will be assessed by a trained, certified investigator at baseline (before treatment) and during the 2nd and 4th weeks of the intervention, as well as at the 8th and 12th weeks of follow-up. A higher score indicates a more severe neurological deficit.

##### Brain magnetic resonance imaging (MRI)

MRI is a non-invasive imaging method to detect features of brain tissue [32]. In particular, MRI is a well-suited technique to visualize and analyse the anatomical properties and abnormalities in the brain. Each participant will be assessed at baseline (before treatment) and during the 4th week of the intervention.

##### Functional magnetic resonance imaging (fMRI)

fMRI is another non-invasive technique that is used to track metabolic activity in the brain. Abnormal fMRI might serve as a potential neuromarker for insomnia [33]. Each participant will be assessed at baseline (before treatment) and during the 4th week of the intervention.

##### Nocturnal concentration of melatonin (MLT)

Melatonin coordinates circadian rhythms and regulates sleep function [34]. Blood samples of patients will be collected at 3:00 a.m. in a dimly lit environment to diminish the impact of light on the secretion of melatonin. Each participant will be assessed at baseline (before treatment) and during the 4th week of the intervention.

#### Safety assessments

Safety will be assessed by renal function tests, liver function tests, routine blood tests, routine urine tests, routine stool tests, and electrocardiograms. These indicators will be detected at baseline (before treatment) and during the 4th week of the intervention.

### Participant timeline {13}

**Fig. 3:**
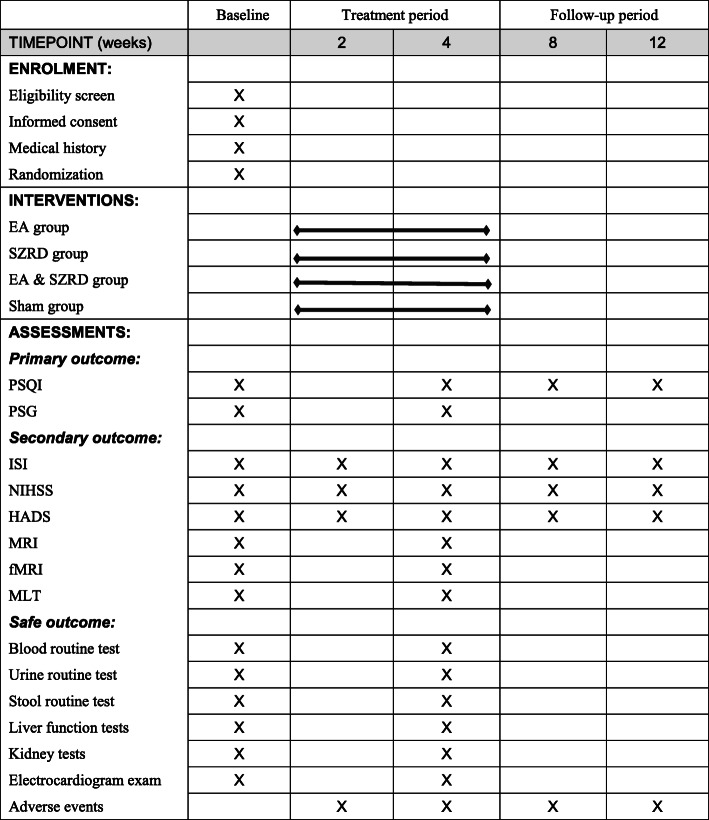
Details of measurements, and time points of data collection. EA, electroacupuncture; SZRD, Suanzaoren decoction; PSQI, Pittsburgh Sleep Quality Index; PSG, polysomnography; ISI, Insomnia Severity Index; NIHSS, National Institutes of Health Stroke Scale; HADS, Hospital Anxiety and Depression Scale; MRI, brain magnetic resonance imaging; fMRI, functional magnetic resonance imaging; MLT, nocturnal concentration of melatonin

Details of the case distribution, specific measurements, and time points of data capture can be found in the SPIRIT Figure (Fig. 3).

### Sample size {14}

PSQI scores will be presented as the primary outcome. The sample size was calculated based on changes in PSQI scores. Based on a previous study [35], the global PSQI score after 4 weeks of treatment was 9.21 ± 4.05 in the intervention group and 13.18 ± 4.32 in the control group. We used the following formula to calculate the sample size.

$$ {n}_1={n}_2=\frac{{\left({Z}_{\alpha }+{Z}_{\beta}\right)}^2\ast 2{\sigma}^2}{\delta^2} $$

On the basis of 0.9 power to detect a significant difference (α = 0.05, two-sided), 25 participants are required for each group. Allowing for a 20% drop-out rate, a sample size of 30 for each group is needed. To improve the RCT quality and reduce the bias, we decided to recruit a sample size of 60 for each group. As a result, we plan to recruit a total of 240 patients for the whole trial.

### Recruitment {15}

Patients with post-stroke insomnia will be recruited through advertisements on the WeChat official platform and hospital bulletin boards. If patients are interested in this study, the research coordinator will obtain their contact details. Then, the principal investigator will make an appointment with each successive patient. In an individual room, the potential participant will be told in detail of the purpose and content of the study as well as the benefits and risks of participating by the principal investigator, who will screen them based on the inclusion and exclusion criteria and obtain informed consent from the eligible patients.

## Assignment of interventions: allocation

### Sequence generation {16a}

The investigator at Yichang Hospital of Traditional Chinese Medicine will generate random numbers 001–240 by SPSS 20.0 software (SPSS Inc., Chicago, IL, USA) and places random numbers in a sequenced, sealed, opaque envelope.

### Concealment mechanism {16b}

The random numbers in the sequenced sealed opaque envelopes will have been equally allocated to the four groups (0, 1, 2, or 3 group). The principal investigator, who will enrol the eligible patients, will open the envelopes based on patients’ registration order numbers and then assign the patients to groups 0, 1, 2, or 3 in accordance with the group number in the envelope. The random numbers will be placed in opaque sealed envelopes to prevent selection bias. The researchers, data collection staff, and data analysts will be blinded in the course of the random allocation sequence to avoid detection bias.

### Implementation {16c}

The designated investigator will generate the allocation sequence, and another investigator designated by the project leader will enrol and assign participants.

## Assignment of interventions: blinding

### Who will be blinded {17a}

We designed a single-blind trial in which the participants of the EA group, the participants of the sham EA group, data collection staff, and data analysts will be blinded during the course of the research. Because of the particular treatment manipulations, blinding the acupuncturists, the participants of the EA & SZRD group, and the SZRD group is impossible. During the trial process, acupuncturists and data collection staff will be forbidden to exchange information with each other and communicate with the study patients. We will conduct each treatment in a closed unit with patients wearing eye masks. To maximize blinding of participants, a sham device will be utilized [36, 37] (shown in Fig. 2A).

### Procedure for unblinding if needed {17b}

Researchers will be permitted to be unblinded in some specific situations, such as participants experiencing serious adverse events or disease progression, and where prompt treatment and appropriate management are needed. The procedure for unblinding will be as follows: (1) prior to unblinding, the researcher will need to inform the trial steering committee. (2) The researcher should unblind the intervention information of the patient based on the emergency envelope. (3) The researcher will need to fill out the report form of unblinding and note it on the case report form (CRF).

## Data collection and management

### Plans for assessment and collection of outcomes {18a}

All data will be documented in detail in the CRF after face-to-face visits. The same researcher will be responsible for the examination of each patient at each assessment. Baseline measurements, medical history, demographics, and previous medications will be collected at the first visit. The primary and secondary outcomes will be assessed at baseline (before treatment) and during the 2nd and 4th weeks of the intervention, as well as at the 8th and 12th weeks of follow-up. Safety assessments will be evaluated at baseline and during the 4th week of the intervention.

### Plans to promote participant retention and complete follow-up {18b}

Follow-up telephone calls and messages on the WeChat public platform will be conducted to promote participant retention.

### Data management {19}

Epidata 3.1 (EpiData Association, Denmark), which is a statistical package, will be used for data entry. Two trained data entry staff members will recheck the CRF and then independently perform data entry. The administrators at Medical College of China Three Gorges University will independently check and judge the input data and confirm whether all CRFs are being completed in a timely manner to ensure that the withdrawal of participants and all adverse events are recorded in CRFs.

### Confidentiality {27}

Patient names in the dataset will be identified with Pinyin initials and numbers. CRFs will be stored in a securely locked location. Epidata 3.1 will be locked and analysed by an independent statistician under the supervision of the administrators. The data and relevant records from this trial will be input on the ResMan Research Manager website at http://www.medresman.org.cn/login.aspx.

### Plans for collection, laboratory evaluation, and storage of biological specimens for genetic or molecular analysis in this trial/future use {33}

No biological specimens will be collected in this trial.

## Statistical methods

### Statistical methods for primary and secondary outcomes {20a}

Based on a previous study, the minimal clinically important difference is approximately 1.14–1.75 points on the PSQI score following treatments [38]. We will assume a treatment response with a score reduction on the PSQI of 2 or more points. Descriptive statistics will be used to analyse baseline characteristics for each group. Repeated measures analysis will be performed to make comparisons between the treatment groups and the control group (EA & SZRD versus sham EA, EA versus sham EA, and SZRD versus sham EA) at different time points (2nd, 4th, 8th, and 12th weeks). If a significant difference is detected, the next step will be to make comparisons among the three treatment groups in effectiveness. The Bonferroni correction method will be used for multiple comparisons. The Kruskal-Wallis test will be used for the analysis of data with a skewed distribution. Analysis of variance will be used for numerical variables, and the chi-squared test will be used for categorical variables. SPSS 20.0 software (SPSS Inc., Chicago, IL, USA) will be used to analyse the data.

### Interim analyses {21b}

Although an interim analysis will not be conducted, the trial should be terminated if there is clear evidence that serious adverse events (SAEs) are associated with the intervention.

### Methods for additional analyses (e.g. subgroup analyses) {20b}

Multivariate logistic regression will be used to conduct exploratory analyses. Based on insomnia duration, insomnia can be categorized into acute insomnia (lasting up to three months) and chronic insomnia (lasting at least 3 months) [39]. A subgroup analysis will be conducted based on the duration of insomnia after stroke.

### Methods in analysis to handle protocol non-adherence and any statistical methods to handle missing data {20c}

The evaluation of the outcomes will be based on an intention-to-treat analysis (ITT). Before statistical analysis of the data, the missing data mechanism will be examined, and the missing data pattern will be investigated. Multiple imputation (MI) is a principled missing data method that provides valid statistical inferences under missing at random conditions [40]. Considering 20% missing data, at least five imputations will be necessary [41]. We will select the preferred imputation method based on the missing data pattern [41].

### Plans to give access to the full protocol, participant-level data, and statistical code {31c}

The datasets generated and analysed during the study will be available from the corresponding author on reasonable request.

## Oversight and monitoring

### Composition of the coordinating centre and trial steering committee {5d}

The coordinating group is composed of the research coordinator and nurses. Nurses are responsible for communicating with patients who are interested in the recruitment advertisement and performing the phone call consultation and WeChat visit of the participants. The research coordinator will help the principal investigator obtain the contact details of potential participants and communicate with patient advisers who provide their experiences as patients with post-stroke insomnia to the design and details of this study. The steering committee (SC) is composed of the subject leader and the project manager. The SC will be responsible for the surveillance of the quality control and the progress of the study. The SC will appoint inspectors as a monitor group to oversee this study in accordance with the Good Clinical Practice guidelines. The monitor group will conduct daily supervision activities and then will write the inspection report, which will mainly include the quality of the CRF form, researchers’ compliance with the protocol, and recruitment processing of patients to the steering committee every week. The SC will have the authority to allow modification of the plan.

### Composition of the data monitoring committee, its role, and reporting structure {21a}

The administrators at the Medical College of China Three Gorges University will be responsible for monitoring data management as an independent third party. The administrators will write the data monitoring report, which will mainly include the accuracy of entries and the data entry staff’s knowledge of various standards, to the SC every week.

### Adverse event reporting and harms {22}

When adverse events (AEs) occur, it will be necessary to record the name of the AE, the beginning and end times of the AE, the outcomes of the AE, the severity of the AE, the treatments related to the AE, causality, and the application of combined drugs. The researchers will analyse causality based on the following five criteria: (1) Is there a reasonable time sequence between the intervention and AE? (2) Is the AE consistent with the type of AEs known to be associated with the intervention? (3) Did the AE disappear or decrease after discontinuation of the intervention? (4) Does the same AE appear when the patient receives the same intervention? (5) Can the AE be explained by a combination of medications or by the patient’s disease progression?

AEs will include nausea, tiredness, dizziness, syncope, palpitations, hepatic dysfunction, and kidney dysfunction. SAE harms include death, life-threatening, hospital treatments, and permanent disability.

### Frequency and plans for auditing trial conduct {23}

Our trial will be audited every 6 months by qualified clinical trial experts, and the review process will be independent of researchers and sponsors.

### Plans for communicating important protocol amendments to relevant parties (e.g. trial participants, ethical committees) {25}

If there is any change to eligibility criteria, outcomes, or analyses, a new version of the protocol will be submitted to the Medical Ethics Committee of College of Traditional Chinese Medicine, China Three Gorges University & Yichang Hospital of Traditional Chinese Medicine, for approval.

## Dissemination plans {31a}

The results of the study will be presented in the form of publications or conference reports.

## Discussion

A stroke occurs when a blood vessel that carries oxygen and nutrients to the brain is either blocked by a clot or bursts (or ruptures), which frequently leads to long-term deficits in physical and cognitive functions in patients [42]. Insomnia is one of the most common complications in stroke patients [43]. Although the exact pathophysiology between insomnia and stroke remains complicated, a basic consensus has been reached that the pathophysiological mechanisms linking insomnia with worse stroke outcomes may be relevant to sympathetic overactivation, hypoxaemia, oxidative stress, and inflammatory reactions [44]. Some studies have provided evidence that insomnia patients show inferior motor function and other worse stroke recovery, and it is believed that healthy sleep plays an important role in the restoration and compensation of neurological functions associated with stroke [45–47]. Ameliorating insomnia during any phase of stroke is positive for short- and long-term recovery [47].

SZRD, one of the most widely used CHM formulas, has been utilized for insomnia for thousands of years in China [19]. A network analysis revealed that the five herbs in SZRD may play different roles in a coordinated manner to exert a range of sedative and hypnotic actions via GABAergic and serotonergic systems [48]. GABAergic activity not only improves sleep but also promotes sleep-related learning and memory processes, which are potentially relevant to neurorehabilitation after stroke [49]. In addition, it has been found that SZRD is beneficial for balancing the nervous system, regulating blood sugar and blood pressure, normalizing the hormone system, and improving the inflammatory response [50].

EA has been used in the treatment of stroke for decades [51]. Recently, it has also been widely utilized as a clinical treatment for insomnia [52]. An evidence-based review showed that EA significantly alleviates pathological damage and promotes post-stroke functional recovery via neurogenesis and astrogliosis [53]. Accumulating data have documented that EA may effectively work by a variety of neurotransmitters, such as adenosine, GABA, galanin, and glutamate, which are related to improving sleep quality [54]. Some clinical RCTs have indicated that EA or EA combined with other therapies is effective for the treatment of insomnia [55, 56].

Therefore, we propose to conduct a clinical RCT on EA plus SZRD for the treatment of post-stroke insomnia. We designed a randomized, four-group, and single-blinded clinical trial with a 4-week treatment period and a 12-week follow-up period. Based on TCM theory and previous studies, *Shenmen* (HT7), *Sanyinjiao* (SP6), *Baihui* (GV20), and *Shenting* (GV24) are the most frequently selected acupoints for the treatment of post-stroke insomnia, and local acupoint stimulation can improve the curative effect when combined with distal acupoints [57, 58]. We also emphasize the experience of the “*de qi*” sensation during the intervention. There have been differences in efficacy between low-frequency and high-frequency electrical stimulation in EA therapy [59, 60]. Accumulating data have shown that low-frequency EA is effective for complications related to stroke, such as insomnia, epilepsy, and pseudobulbar palsy [61–63]. We selected a 10-Hz frequency of EA to treat insomnia after stroke in this trial. Preliminary analyses have indicated that different types of sham acupuncture may have different effect sizes, and a meta-analysis showed that sham devices may have minimum non-specific effects [64, 65]. We chose to apply a sham device in the sham group to ensure the precision of the results.

Non-invasive neuroimaging technology is the most commonly recommended diagnostic tool in stroke [66]. Technologies such as fMRI open a window for the study of insomnia after stroke. Some researchers have found that patients with primary insomnia have abnormal brain metabolism and connectivity related to particular brain regions, such as the prefrontal cortex, insular cortex, amygdala, precuneus, and caudate [67, 68]. Thus, we can utilize fMRI to assess aspects of treatment-induced changes in resting-state brain metabolism and elucidate the possible underlying mechanisms of these therapies in this protocol.

Melatonin is a hormone secreted by the pineal body, with peak secretion at night [69]. Some researchers have found that abnormal secretion of melatonin is potentially related to insomnia in stroke patients [70, 71]. A preliminary report revealed that acupuncture increased nocturnal melatonin secretion and reduced insomnia and anxiety [72]. We will detect the nocturnal melatonin concentrations in patients with post-stroke insomnia to investigate whether EA plus SZRD can also play a beneficial role by increasing nocturnal melatonin secretion and reducing insomnia.

We hope to elucidate the possible underlying mechanisms of this combination therapy by using MRI and melatonin detection. However, our study has some limitations. One limitation is the use of self-rating scales, which might affect the evaluation of the severity of the insomnia disorder. However, we will attempt to decrease the subjective factors by PSG. Another limitation is that the trial will be performed in a single-blind manner, and it will be conducted in only one medical centre. These limitations may weaken the generalizability of the study. A large sample, multicentre, study using objective measures to assess the efficacy of EA plus SZRD treatment should be conducted in a future study.

## Trial status

Recruitment began in April 2020 and the approximate date when recruitment will be completed is January 2022. Protocol version 2.0 was approved on January 16, 2020.

## Abbreviations

EA: Electroacupuncture
SZRD: Suanzaoren decoction
TCM: Traditional Chinese medicine
CHM: Chinese herb medication
PSG: Polysomnography
PSQI: Pittsburgh Sleep Quality Index
ISI: Insomnia Severity Index
HADS: Hospital Anxiety and Depression Scale
NIHSS: National Institutes of Health Stroke Scale
MRI: Brain magnetic resonance imaging
fMRI: The functional magnetic resonance imaging
MLT: Nocturnal concentration of melatonin
RCT: Randomized controlled trial
CRF: Case report form
